# Olanzapine-induced methylation alters cadherin gene families and associated pathways implicated in psychosis

**DOI:** 10.1186/1471-2202-15-112

**Published:** 2014-09-29

**Authors:** Melkaye G Melka, Christina A Castellani, Nagalingam Rajakumar, Richard O’Reilly, Shiva M Singh

**Affiliations:** Molecular Genetics Unit, Department of Biology, The University of Western Ontario, London, ON N6A 5B7 Canada; Department of Psychiatry, The University of Western Ontario, London, ON N6A 5B7 Canada

## Abstract

**Background:**

The complex aetiology of most mental disorders involves gene-environment interactions that may operate using epigenetic mechanisms particularly DNA methylation. It may explain many of the features seen in mental disorders including transmission, expression and antipsychotic treatment responses. This report deals with the assessment of DNA methylation in response to an antipsychotic drug (olanzapine) on brain (cerebellum and hippocampus), and liver as a non-neural reference in a rat model. The study focuses on the Cadherin/protocadherins encoded by a multi-gene family that serve as adhesion molecules and are involved in cell-cell communication in the mammalian brain. A number of these molecules have been implicated in the causation of schizophrenia and related disorders.

**Results:**

The results show that olanzapine causes changes in DNA methylation, most specific to the promoter region of specific genes. This response is tissue specific and involves a number of cadherin genes, particularly in cerebellum. Also, the genes identified have led to the identification of several pathways significantly affected by DNA methylation in cerebellum, hippocampus and liver. These included the Gα12/13 Signalling (*p* = 9.2E-08) and Wnt signalling (*p* = 0.01) pathways as contributors to psychosis that is based on its responsiveness to antipsychotics used in its treatment.

**Conclusion:**

The results suggest that DNA methylation changes on the promoter regions of the Cadherin/protocadherin genes impact the response of olanzapine treatment. These impacts have been revealed through the identified pathways and particularly in the identification of pathways that have been previously implicated in psychosis.

## Background

Cadherins are involved in neurodevelopment, nerve cell migration and cell-to-cell interaction and therefore it follows that they may regulate the pathogenesis of psychosis [1]. For example, previous studies suggested the importance of investigating epigenetic control of *PcdhXY* with respect to psychiatric disorders [2]. Interestingly, previous studies have suggested that epigenetic modifications regulate the expression of clustered protocadherin genes (*Pcdhs*) [3]. In mammals, more than 50 *Pcdh* genes are categorized into three clusters *Pcdh-α, Pcdh-β* and Pcdh-γ encoding diversified proteins mainly expressed in the nervous system [3–5]. Some protocadherins (*Pcdh1* and *Pcdh7*) are regulated by Methyl-CpG-binding protein, *MeCP2*, which is a protein that is strongly associated with epigenetic regulation [6].

Aberrant promoter methylation of cadherin family genes has been implicated in complex human disorders [7]. For example, *Pcdh10*, a novel tumor suppresser gene is found to be downregulated by hypermethylation in various cancer cases [8–10] corroborating the evidence that epigenetics plays a role in complex disorders. Further, the high expression of *Pcdhαv8* in mouse neuroblastoma cell lines was found to be associated with demethylation of the 5’ regulatory region of the gene [11]. In the same study, a dynamic change in methylation of Pcdh genes was observed. The 5’ region of the *Pcdhαv4* gene was 99% methylated in neuroblastoma cells and ~35% methylated in the cortex of mice. On the contrary, the 5’ region of the *Pcdhαv8* was reported to be 99.1-100% methylated in neuroblastoma cell lines and 5% methylated in the cortex of the mouse strain C57BL/6 [11].

Although it is possible that aberrant metabolomics may be due to mutation in a single gene or be the effect of a single environment, emerging literature argues that most often the development of complex diseases and disorders represents a complex interaction of multiple genes and multiple environments [12]. This is true in most behavioral disorders that have complex aetiology involving several genes and environment [13]. Ultimately, the two contributors enact their effect by altering cellular metabolomics through changes in gene expression via epigenetic modifications such as DNA methylation [14]. However, an assessment of the mechanistic basis of the effect of environment has been arduous as an environment may cause aberration via its effect on the gene(s). This offers renewed hope for the treatment of complex disorders, as it may be possible to manipulate DNA methylation, and subsequently alter gene expression to affect metabolomic aberrations and facilitate a cure or decreased severity of symptoms. Also, this paradigm of disease causation is expected to be instrumental in identifying mechanisms of actions of antipsychotics, such as olanzapine.

Mental disorders including psychosis are difficult to treat. Antipsychotics have variable responses and their mechanism of action is imprecise. We have assessed the mechanisms of action of olanzapine, a commonly used antipsychotic medication. The results argue that olanzapine may improve the mental outcome of patients by altering the methylation profile of genes implicated in disease-specific pathways. Specifically, our studies reported changes in the methylation of genes implicated in the dopamine pathways in the hippocampus and cerebellum in a rat model [15]. Interestingly, in a genome-wide assessment, our studies showed methylation changes in the genes of a number of pathways that may alter metabolomics leading to the efficacy as well as side effects of olanzapine [15]. These results offer a novel paradigm to reveal critical genes and pathways by identifying changes in methylation in response to drugs that offer relief or cure for the disease symptoms. Here we will present data on genes of the cadherin and protocadherin (*Pcdh*) family that are preferentially affected by olanzapine. The clustered protocadherin (*Pcdh*) genes [16] encode a group of diverse cadherin-related transmembrane proteins, and are expressed in the brain [17]. They generate vast single-cell diversity in the brain [18]. They represent Ca_2_^+^-dependent cell adhesion molecules that are critical in vertebrate nervous development [19] and maintenance of neural circuitry [20]. The expression of this cluster of genes is regulated by a complex mechanism at the level of transcription [21] that may include gene specific DNA methylation [22]. Given the critical role of cadherins in brain development and function it is not surprising that genome-wide analysis for a number of mental disorders have implicated defects in cadherin genes. These include complex disorders, such as intellectual disability [23] autism [24] schizophrenia [25, 26] and bipolar disorders [27]. This report adds to this complex literature suggesting that a commonly used antipsychotic drug, olanzapine, affects methylation of the promoter regions of cadherin family genes in the rat brain. The effect of olanzapine is tissue specific, particularly affecting several gene promoters in cerebellum and may have implications in the therapeutic response.

## Methods

### Rats

Adult male Sprague-Dawley rats of 12 weeks of age (250 - 300 g) were purchased from Charles River, PQ, Canada. Upon arrival, rats were placed (2 per cage) in standard acrylic cages and subjected to the treatment protocol. They housed in controlled humidity and temperature on a 12-hour light/dark cycle (lights on at 7:00 a.m.). They were fed standard rat chow (LabDiet) and tap water *ad libitum.*

### Ethical approval

The Institutional Animal Care Committee of the University of Western Ontario had approved all animal-related procedures used in this study following the Canadian and National Institute of Health Guides on animal experimentation. Also, the manuscript reporting followed the ARRIVE (Animal Research: Reporting of *In Vivo* Experiments) guidelines.

### Olanzapine treatment

Before the commencement of olanzapine treatment, animals were weighed and divided into two treatment groups with comparable means of weight. Their stress-induced locomotor activity (following a 5 min tail pinch) was recorded for 30 min using an automated open-field activity chamber (San Diego Instruments, San Diego, CA, USA). A computer that detects the disruption of photocell beams recorded the number of beam breaks per five minutes for half an hour as the animal moves. Rats then received injections of olanzapine (Zyprexa, Lilly, IN, USA; 2.5 mg/kg, i.m.; n = 8) or vehicle (phosphate buffered saline (PBS); n = 8) between 1:30 pm and 3:00 pm daily for 19 days. On day 20, eighteen hours after the last olanzapine/vehicle injection, rats were subjected to stress-induced locomotor activity to assess the therapeutic efficacy of chronic olanzapine. Significantly reduced locomotor activity of olanzapine treated rats indicated the therapeutic efficacy of the drug administered, which was comparable to the dosing paradigm employed in previous studies [28].

### Genomic DNA extraction

Rats were decapitated without anaesthesia, brain tissues were micro-dissected promptly in ice-cold PBS; and three random biopsy punches through cerebellum, hippocampus, and liver were obtained. Individual samples (three random biopsy punches of each tissue composed one sample) from each rat were kept separately and flash frozen in liquid nitrogen. The genomic DNA was isolated from olanzapine-treated (*n* = 2) and saline control (*n* = 2) samples of cerebellum, hippocampus and liver. Genomic DNA was isolated from the interphase layer of TRIzol using sodium citrate, followed by ethanol precipitation and purification using the QIAamp® DNA Micro Kit (QIAGEN, Valencia, CA). DNA was then quantified using a NanoDrop ND-1000 spectrophotometer (Thermo Fisher Scientific Inc., Wilmington, DE) and all samples had OD_260_/OD_280_ nm ratios of 1.8–2.0 and OD_260_/OD_230_ nm ratios of 2.0–2.4.

### Methylated DNA immunoprecipitation

This study has used methylated DNA immunoprecipitation (MeDIP) to investigate genome-wide DNA methylation in a rat model [29] The MeDIP sample labeling, hybridization, and processing were performed at Arraystar Inc. (Rockville, Maryland, USA). The immunoprecipitated DNA was eluted and purified by phenol chloroform extraction and ethanol precipitation. The total input and immunoprecipitated DNA were labeled with Cy3- and Cy5-labeled random 9-mers, respectively, and hybridized to NimbleGen RN34 Meth 3×720 K CpG plus Promoter array, which is a single array design containing all known CpG Islands annotated by UCSC and all well-characterized RefSeq promoter regions (from about -3.88 kb to +0.97 kb of the transcription start sites [TSSs]) covered by ~720,000 probes. Scanning was performed with the Axon GenePix 4000B microarray scanner. Therefore, the array design would capture the global changes in methylation.

Data analysis involved the comparison of differentially enriched regions between drug exposed (E) and control (C) rats, the log_2_-ratio values were averaged and then used to calculate the M’ value [M’ = Average (log_2_ MeDIPE/InputE) – Average (log_2_ MeDIPC/InputC)] for each probe. A NimbleScan sliding-window peak-finding algorithm was run on this data to find the differential enrichment peaks (DEP). Using an R script program, a hierarchical clustering analysis was completed. The probe data matrix was obtained by using PeakScores from differentially methylated regions selected by DEP analysis. This analysis used a “PeakScore” ≥2 to define the DEPs, which is equivalent to the average p-value ≤0.01, for all probes within the peak. In this study, we have specifically analyzed 17 Cadherin family genes and their methylation status in cerebellum and hippocampus with liver as a non-neural cell types following olanzapine treatment of experimental rats (Table 1).

**Table 1:** **List of the Cadherin genes with their promoter regions affected by olanzapine-induced methylation**

Gene name	C	Olz	Chr	Strand	TSS	TTS	Peak to TSS	Accession
**Cerebellum**								
*Pcdh7*	+	-	14	-	57323892	56609035	-3782	NM_001004087
*Pcdh8*	-	+	15	-	61060741	61056993	-252	NM_022868
*Pcdh9*	-	+	15	-	76737306	75827179	-2160	NM_001191688
*Pcdha11*	+	-	18	+	29728602	29928030	-1925	NM_199486
*Pcdha9*	-	+	18	+	29715234	29928031	-3346	NM_199508
*Pcdhga5*	+	-	18	+	30600857	30754205	914	NM_001037137
*Pcdh19*	-	+	X	-	121209099	1.21E + 08	-777	NM_001169129
*Cdh1*	+	-	19	+	36442692	36512091	-92	NM_031334
*Cdh13*	-	+	19	+	48507172	49575122	-501	NM_138889
*Pcdh18*	+	-	2	-	138629181	1.39E + 08	-3583	NM_001100524
*Cdh22*	+	-	3	-	156275540	1.56E + 08	94	NM_019161
**Hippocampus**								
*Pcdhga8*	+	-	18	+	30635032	30754205	-3889	NM_001037156
*Cdh17*	-	+	5	+	26047221	26099173	-1218	NM_053977
*Cdh23*	-	+	20	-	28016145	27622048	-214	NM_053644
*Pcdh7*	-	+	14	-	57323892	56609035	-1007	NM_001004087
**Liver**								
*Pcdhgb7*	+	-	18	+	30660279	30754207	-1029	NM_001012215
*Cdh11*	-	+	19	+	2225208	2382805	923	NM_053392
*Cdh5*	-	+	19	-	816108	779349	-46	NM_001107407
*Pcdhga8*	+	-	18	+	30635032	30754205	-3889	NM_001037156

C: Control; Olz: olanzapine treated; Chr: chromosome; +: increase in DNA methylation; -: decrease in DNA methylation.

### Pathway analysis of array results

The affected Cadherin family genes were then subjected to ingenuity pathway analysis (Ingenuity System Inc, CA, USA) [http://www.ingenuity.com/]. Transcription factor binding sites of *Cdh13* that showed increased methylation in cerebellum were identified using CTCFBSDB 2.0 [30]. This analysis was done as a representative for all cadherin genes affected by olanzapine-induced DNA methylation.

## Results

Recently, we reported that therapeutic olanzapine causes an increase in 1294 genes and a decrease in 565 genes in the cerebellum of treated rats [15]. This response is tissue specific and only 10% of the genes affected were shared between cerebellum and hippocampus. With respect to cadherin genes, we report that the promoter regions of 11 Cadherin family genes have olanzapine-induced DNA methylation changes in the cerebellum as compared to matched controls (Table 1). Of those genes, five showed significantly increased methylation, while six of them showed decreased methylation (*p* < 0.01). In comparison to the results on cerebellum, three cadherin genes (*Cdh17, Cdh23, Pcdh7*) had increased methylation and one gene (*Pcdhga8*) showed decreased methylation in hippocampus. Also, two cadherin genes (*Cdh5* and *Cdh11*) had increased methylation and two genes (*Pcdhga8* and *Pcdhgb7*) showed decreased methylation in the liver. The cadherin genes in the rat genome that are affected by olanzapine-induced DNA methylation are dispersed over different chromosomes including chromosome 2, 3, 15, 18, 19, 20 and X.

**Figure 1:**
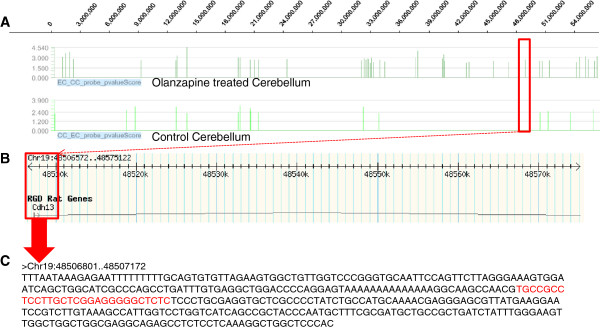
**A representative figure depicting the genomic region of Cadherin 13 gene affected by olanzapine. A)** Manhattan plot showing the significance of methylation enriched regions. Red box shows DNA methylation enriched region containing subset of CpG sites overlapping with the promoter region of *Cdh13* on Chromosome 19, in olanzapine treated cerebellum compared to control (upper panel), while there was no methylation enriched subset of CpGs at that particular genomic region in the control cerebellum compared to treated cerebellum sample (lower panel) **B)** Red box shows the gene name and the promoter region affected by olanzapine-induced DNA methylation, obtained from Rat Genome Database, **C)** Part of the promoter sequence entirely affected by olanzapine-induced DNA methylation, and the CTCF binding site sequence is highlighted in red.

For the identification of each methylation difference, we compared the Manhattan plot of individual cadherin genes. It is shown in Figure 1 using *Cdh13* as a model. In this figure, the peak of differential methylation (Figure 1A), the genomic region of *Cdh13* on the rat genome (Figure 1B) and the sequence depicting the promoter region (Figure 1C) are shown. It has allowed us to identify regulatory features including a CTCF binding site. As shown in Figure 1C, the methylation changes were observed in the surrounding Cs and CpGs suggesting the global effect of olanzapine. The sequence presented in the figure showed differential methylation, which included the identified CTCF binding site in the promoter region of the gene.

**Figure 2:**
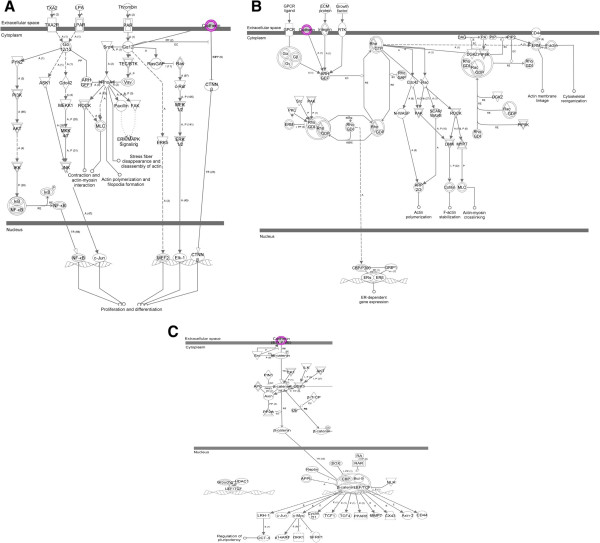
**The most significant pathways identified by Ingenuity pathway analysis. A)** Gα12/13 signalling (*p* = 9.2E-08), **B)** RhoGDI signalling (p = 4.8E-07), **C)** Wnt/β-catenin signalling (*p* = 1.2E-02) significantly affected by DNA methylation of the Cadherin genes.

Next, we used pathway analysis to identify major pathways and systems (Table 2) that may be affected by the observed olanzapine-induced methylation changes in the cadherin family genes in hippocampus, cerebellum and liver listed in Table 1. Interestingly, pathway analysis involving these genes revealed the most significant pathways including the Gα12/13 signalling (*p* = 9.2E-08) (Figure 2A), RhoGDI signalling (p = 4.8E-07) (Figure 2B) and Wnt/β-catenin signalling (*p* = 1.2E-02) pathways (Figure 2C). They affect molecular and cellular functions associated with cell-to-cell signalling and interactions (*p* = 6.13E-04 - 3.01E-02) and may affect tissue, nervous system dynamics and behaviour. In doing so, differential methylation of genes involved in the pathway suggested that olanzapine affects the physiological cadherin system (*p* = 6.13E-04 - 9.76E-03) (Table 2).

**Table 2:** **Top pathways, networks and molecular and cellular functions identified by pathway analysis using the Cadherin family genes affected by olanzapine**

Top canonical pathways	*P*-value	Number of molecules/ratio*
Gα12/13 Signalling	9.16E-08	5/113 (0.044)
RhoGDI Signalling	4.76E-07	5/166 (0.03)
Signalling by Rho Family GTPases	2.22E-06	5/221 (0.023)
Wnt/β-catenin Signalling	1.25E-02	2/158 (0.013)
Thyroid Cancer Signalling	4.16E-02	1/40 (0.025)
**Molecular and Cellular Functions**		
Cell-to-cell Signalling and interaction	6.13E-04 - 3.01E-02	4
Cellular movement and development	1.09E-03 - 2.37E-02	4
**Physiological System Development and Function**		
Behaviour, tissue development, nervous system development and function	6.13E-04 - 9.76E-03	5
**Top Networks**		
Cellular Movement, Cellular Growth and Proliferation, Cancer		5

*For the top canonical pathways, ratio shows the number of molecules in a given pathway that suffice the DNA methylation status cut-off (p<=0.01) divided by the total number of molecules in the pathway.

## Discussion

Cell-cell communication is an essential feature of multicellular organisms. It is particularly critical in the mammalian brain, where molecules involved in cell-cell interactions such as adhesion and exchange of information provide the foundation for neurodevelopment as well as proper functioning of the neural network. Not surprisingly, mammalian brain is rich in such molecules. Also, the exceptional richness and diversity of these molecules is often accounted for by their evolution, by multi-gene families and by diversity of regulatory mechanisms. For example, adhesion molecules such as cadherins that constitute a super-family of transmembrane receptors and mediate Ca^2+^ -dependent cell to cell hemophilic interactions are encoded by ~100 genes in humans [31]. Also, the organization of these genes allows differential expression including gene specific DNA methylation and differential splicing to facilitate expression of specific cadherins in different cells and cell types [32]. As it stands, the molecular basis of the cadherins is still unclear. What is known is that cells expressing a given cadherin adhere preferentially to those expressing the same cadherin subtype [33]. Also, the combination of qualitative and quantitative differences likely confers cell type-specific adhesion affinities on individual cells [34]. They provide the foundation for target recognition, synapse formation and signalling, which are critical features of any neural network [35].

The results included in this study show that olanzapine affects the methylation status of the cadherin gene promoters. Given the involvement of cadherin genes in a number of mental disorders including psychosis, it is understandable that several cadherin genes are differentially methylated in response to olanzapine. Also, this difference is prominent in the cerebellum that is involved in cognitive processing and emotional control including attention, executive control, language, working memory, pain, emotion, and addiction in addition to its role in motor coordination [36]. The results of this study have identified that the methylation differences are specific to the promoter region of individual genes (Table 1). Also, the sequence features of these regions contain regulatory signals (Figure 1) and cellular metabolomics. The associated cellular changes are compatible with anomalies in behavioral disorders via three specific pathways: Gα12/13 signalling, RhoGDI signalling and Wnt signaling (Figure 2). Gα12/13 are units of heterotrimeric G proteins that regulate cell processes including cell remodeling through the use of guanine nucleotide exchange factors [37] while Rho family proteins regulate a wide diversity of cellular processes, including adhesion, cytokinesis, cell-cell progression, membrane trafficking and signal transduction [38]. Interestingly, the Wnt signaling pathway has been implicated in the pathogenesis of schizophrenia [39] as well as negative symptom severity in psychosis [40] and it has been previously shown that E-cadherin and β-catenin affect Wnt Signaling pathway [41]. Further, aberrant expression of transcripts of the *PCDH11*X/Y gene was reportedly implicated in anomalous cerebral asymmetry [42]. This has led to the possibility that the variation relating to psychosis and psychological disorders may not only be sequence based, but may also be mediated by antipsychotic-induced epigenetic changes [39, 42]. The results on olanzapine-induced differential methylation of cadherin genes implicated in the three pathways add further support to this still evolving paradigm. Of special interest is the *Pcdh8* gene. It has been implicated in schizophrenia [20] and has increased methylation both in hippocampus and liver in this study, suggesting that the expression of the gene is likely to be compromised due to the antipsychotic drug. The observed hypermethylation of the gene could potentially repress its transcription [22]. Moreover, there are indications that epigenetic changes affect not only a single candidate gene promoter but also intragenic sequences located farther from the transcriptional start sites [43]. The observed methylation changes are tissue-specific, and it is noted that the direction of methylation change (either increased or decreased methylation) is not consistent across tissues, suggesting the possibility that the changes in brain tissues are likely related to the efficacy of the drug while effects in liver may be related to the adverse consequences of the drug. Olanzapine-induced DNA methylation changes in genes involved in the identified pathways may alter the associated network functions (Table 2). However, further study is required to specifically analyze the effects (on a protein level) of gene-specific methylation changes on each identified network to unravel the specific mechanism of action of olanzapine. Also, we acknowledge that the prefrontal cortex and nucleus accumbens that are also implicated in psychosis [44, 45] may need to be investigated for the effect of antipsychotics on methylation, in future studies.

## Conclusion

The identified pathways suggest that olanzapine-induced DNA methylation changes on the promoter regions of the Cadherin/protocadherin genes may have an important role in response to olanzapine treatment in psychosis. Further, the approach outlined in this study may be applied to other disorders of complex aetiology.
